# β–Cyclodextrin–Propyl Sulfonic Acid Catalysed One-Pot Synthesis of 1,2,4,5-Tetrasubstituted Imidazoles as Local Anesthetic Agents

**DOI:** 10.3390/molecules201119696

**Published:** 2015-11-12

**Authors:** Yan Ran, Ming Li, Zong-Ze Zhang

**Affiliations:** 1 Zhongnan Hospital of Wuhan University, Department of Anesthesiology, Wuhan University, Wuhan 430071, China; ranyanqwe@sina.com; 2 Department of Doppler Ultrasound, PLA421 Hospital, Guangzhou 510318, China; liming8782@sina.com

**Keywords:** synthesis, imidazoles, local anesthetic agent

## Abstract

Some functionalized 1,2,4,5-tetrasubstituted imidazole derivatives were synthesized using a one-pot, four component reaction involving 1,2-diketones, aryl aldehydes, ammonium acetate and substituted aromatic amines. The synthesis has been efficiently carried out in a solvent free medium using β-cyclodextrin-propyl sulfonic acid as a catalyst to afford the target compounds in excellent yields. The local anesthetic effect of these derivatives was assessed in comparison to lidocaine as a standard using a rabbit corneal and mouse tail anesthesia model. The three most potent promising compounds were subjected to a rat sciatic nerve block assay where they showed considerable local anesthetic activity, along with minimal toxicity. Among the tested analogues, 4-(1-benzyl-4,5-diphenyl-1*H-*imidazol-2-yl)-*N*,*N*-dimethylaniline (**5g**) was identified as most potent analogue with minimal toxicity. It was further characterized by a more favourable therapeutic index than the standard.

## 1. Introduction

Due to the ease of modification, diversity and effective synthetic options, heterocyclic compounds were considered as the preferred choice for the discovery of novel medications [1]. Of the many varieties available, compounds containing imidazole moieties have attracted considerable attention from medicinal chemists due to their wide array of medicinal properties [2], as it presents diverse pharmacological activities such as antimalarial [3], antibacterial [4], antihistaminic [5], anti-inflammatory [6], analgesic [7], antitubercular [8], antiprotozoal [9] and anthelmintic effects [10]. On the other hand, it has also found a special place in green chemistry as an ionic liquid component [11].

Therefore, extensive work has been carried on the development of efficient ways to prepare imidazoles. Particularly, four component syntheses involving condensation of a 1,2-diketone, a hydroxyketone or a ketomonoxime with an aldehyde, primary amine and ammonium acetate using HY zeolite [12], silica gel/NaHSO_4_ [13], HClO_4_–SiO_2_ [14], molecular iodine [15], BF_3_–SiO_2_ [16], InCl_3_·3H_2_O [17] or potassium dodecatugstocobaltatetrihydrate (K_5_CoW_12_O_40_·3H_2_O) [18] as catalysts afforded 1,2,4,5-tetrasubstituted imidazoles in excellent yield. More recently, Ziarani *et al*., have reported the synthesis of 1,2,4,5-tetrasubstituted imidazoles in excellent yield under solvent free conditions using sulfonic acid functionalized silica (SiO_2_-Pr-SO_3_H) as a catalyst [19]. However, the existing methods are not effective and are associated with several drawbacks, which include long reaction times, harsh reaction conditions, low yields of the products, tedious work-up procedures, and the use of toxic reagents.

**Scheme 1:**
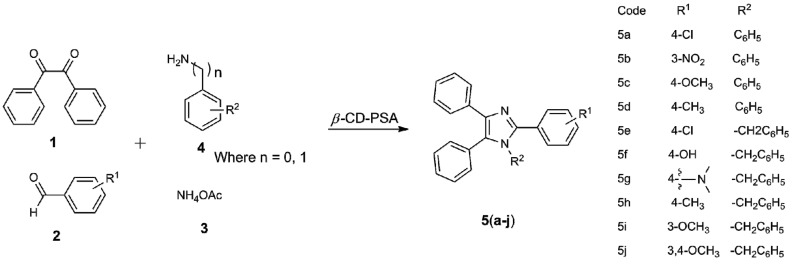
One pot synthesis of 1,2,4,5-tetrasubstituted imidazoles catalysed by β-CD-PSA. Where *n* = 0 means no spacer methylene fragment, *i.e.*, aniline (**5a**–**5d**); *n* = 1 means having the methylene spacer, *i.e.*, benzylamine (**5e**–**5j**).

Herein, we provide a green and economical one-pot synthesis of some 1,2,4,5-tetrasubstituted imidazoles catalysed by β-cyclodextrin–propyl sulfonic acid (β-CD-PSA) as catalyst, as depicted in Scheme 1. The products were also tested for local anesthetic activity using the rat tail flick and corneal hyperflexion methods.

## 2. Results and Discussion

### 2.1. Chemistry

**Scheme 2:**
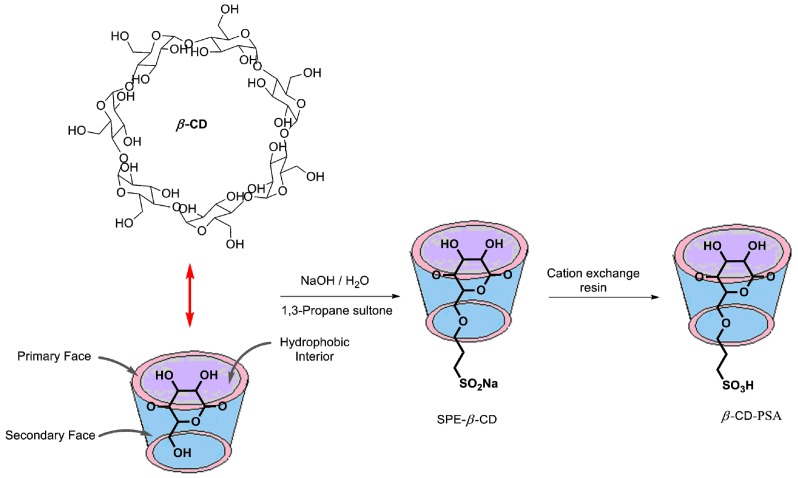
The synthesis of β-CD-PSA.

The synthesis of β-CD-PSA is shown in Scheme 2. Initially, the commercially available β-CD was allowed to react with 1,3-propanesultone in NaOH solution to furnish sulfopropyl ether β-cyclodextrin (SPE-β-CD). This was further treated with an acidic resin to afford β-CD-PSA. The identification of β-CD-PSA was performed via spectroscopic methods and elemental analysis. The average degree of substitution in β-CD-PSA was estimated from ^1^H-NMR spectroscopy, and it was about 4.8. Moreover, the loading efficiency of propylsulfonic acid per g catalyst was also determined by elemental analysis as 2.45 mmol/g.

Next, to optimise the reaction conditions, initially the condensation reaction was examined using benzil (1 mmol), 4-chlorobenzaldehyde (1 mmol), ammonium acetate (3 mmol) and the catalyst β-CD-PSA to afford compound **5a** (Scheme 1). As evident from Table 1, in the absence of catalyst at room temperature, the reaction does not proceed. Further, no change in reaction behaviour was noted on increasing the temperature. However, on introduction of the catalyst, the reaction proceeds well at room temperature to yield compound **5a**. Significant improvement in product yield was reported on increasing the temperature up to 100 °C, keeping the catalytic load constant, *i.e.*, at 50 °C, 80 °C, and 100 °C, the yield of product obtained is 55%, 85% and 96%, respectively. This result suggests the role of catalyst and temperature for the completion of the reaction. In order to further confirm the role of β-CD-PSA, the next set of reactions (entries 7 and 8) was carried out at higher and lower catalyst concentration than 2 mol %. To our surprise, a significant decline in product yield was observed in both the cases, along with longer time being required for completion of the reaction. No further improvement in product yield was seen in water as well as in other solvents. It has thus been corroborated that the maximum yield of the product was obtained under solvent free conditions at 80 °C with a catalytic load of 2 mol % of β-CD-PSA.

**Table 1:** Optimisation of reaction conditions for the synthesis of **5a** ^a^.

Entry	Solvents	Temp (°C)	Amount (mol %)	Time (min)	Yield (%) ^b^
1	No Solvent	rt.	--	150	No reaction
2	No Solvent	80	--	150	No reaction
3	No Solvent	rt.	2	150	28
4	No Solvent	50	2	40	55
5	No Solvent	80	2	30	85
6	No Solvent	100	2	20	96
7	No Solvent	80	1	70	74
8	No Solvent	80	3	50	92
9	Water	80	2	30	63
10	CH_3_CH_2_OH	reflux	2	40	78
11	ClCH_2_CH_2_Cl	reflux	2	40	45
12	EtOAc	reflux	2	40	70
13	THF	reflux	2	40	56
14	CH_3_CN	100	2	40	64
15	DMF	100	2	40	72

^a^ Reaction conditions: benzyl (1 mmol), aromatic aldehyde (1 mmol), ammonium acetate (3 mmol), catalyst β-CD-PSA, solvent free or with solvent (5 mL); ^b^ Isolated yield.

The next part of the study was aimed at comparing the efficiency of β-CD-PSA with other reported catalysts for the synthesis of 1,2,4,5-tetrasubstituted imidazoles. The results are shown in Table 2. It was found that the presence of catalyst together with high heat leads to the completion of the reactions to afford the target derivatives in considerable yield, accompanied by variations in the reaction time. Thus, it could be suggested that β-CD-PSA acts as a better catalyst in respect of reaction times and yields of the products than other previously used catalysts.

After successful optimisation of the reaction conditions, the next part of the work was focused on examining the versatility and limitations of this method using diverse substituents (Table 3). In the majority of the cases the reaction proceeds smoothly, for instance, compound **5a** was furnished in the highest yield among all the derivatives. In the case of compound **5b**, the time required to complete the reaction was increased significantly, along with a minor reduction in yield. In general the reaction time was further increased in the case of compounds bearing electron withdrawing substituents *i.e.*, entries 3 and 4. It is noteworthy to mention that, the yield of the product was decreased significantly along with extended reaction times with the introduction of a methylene bridge to connect phenyl at R_2_. Thus, it has been inferred that derivatives containing phenyl without the spacer were well suited to this condensation reaction to afford 1,2,4,5-tetrasubstituted imidazoles with varying reaction times and yields. On further comparing the results of these derivatives with the methods reported earlier (*cf.* Table 2), it has been found that a variety of catalysts were used for the synthesis of the target compounds. The data presented in Table 2 suggests that, in the majority of cases, the reaction was carried out at higher temeperatures together with use of polar solvents such as ethanol, methanol, acetic acid, DMF and DMSO, leading to complex isolation and recovery procedures. Additionally, these processes generate waste containing catalyst and solvent, which have to be recovered, treated and disposed off. Moreover, the use of microwave irradiation prevents the synthesis of these compounds in resource-poor laboratories because of its expensive instrumentation. In this regard, the present methodology offers advantages over these other methods and furnisheds the desired compounds using greener aspects of efficient conditions without extensive purification. The structures of all newly compounds were ascertained on the basis of IR, ^1^H-NMR, ^13^C-NMR analysis, mass and elemental analysis and correlated with established M.P. reported in previous literatures as given in Table 3 (melting point).

**Table 2:** The comparison of efficiency of various catalysts for the synthesis of 1,2,4,5-tetrasubstituted imidazoles ^a^.

Entry	Catalyst	Solvent	Condition	Yield (in %) ^b^	Time	Ref.
1	Zeolite HY	-	MW	42–85	6 min	[12]
2	Silica gel	-	MW	60–90	6 min	[12]
3	Silica gel/NaHSO_4_	DCM	reflux	85–90	2.5 h	[13]
4	HClO_4_–SiO_2_	-	140 °C	60–94	15–20 min	[14]
5	I_2_	Ethanol	75°C	97–99	15–20 min	[15]
6	K_5_CoW_12_O_40_·3H_2_O	DCM	140 °C	15–95	2.5 h	[8]
7	BF_3_·SiO_2_	-	140 °C	80–96	2 h	[16]
8	InCl_3_·3H_2_O	MeOH	r.t.	47–84	6–9 h	[17]
10	[(CH_2_)_4_SO_3_HMIM]	-	140 °C	85–95	2–2.5 h	[20]
11	MCM-41	-	140 °C	74–82	1.92–2.25 h	[21]
12	MCM-41	AcOH	reflux	75–85	25–35 min	[21]
13	*p*-TsOH	-	140 °C	75–82	1.92–2.17 h	[21]
14	*p*-TsOH	EtOH	reflux	73–83	13–23 min	[21]
15	-	1-Butyl-3-methylimidazolium bromide	140 °C	82–93	1.5–5 h	[22]
16	-	1-Butyl-3-methylimidazolium bromide	MW	82–93	3–8 min	[22]
17	P_2_O_5_/SiO_2_	-	100 °C	87–98	15–55 min	[23]
18	-	-	140 °C	0	3 h	[20]
19	SiO_2_-Pr-SO_3_H	-	140 °C	85–98	10 min–3 h	[19]
20	β-CD-Pr-SO_3_H	-	100 °C	68–96	15 min–4 h	(This work)

^a^ Reaction condition: benzil (1 mmol), aromatic aldehyde (1 mmol), ammonium acetate (3 mmol), catalyst β-CD-PSA (0.02 mmol), 100 °C. ^b^ Isolated yield.

**Table 3:** Synthesis of various 1,2,4,5-tetrasubstituted imidazoles catalysed by β-CD-PSA under solvent free conditions ^a^ [20,21,22,23,24].

Entry	R_1_	R_2_	Product	Time	Yield (in %) ^b^	M.P. in °C (Observed)	M.P. in °C (in Literature)
1	4-Cl	C_6_H_5_	**5a**	30 min	96	158–160	157–159
2	3-NO_2_	C_6_H_5_	**5b**	15 min	93	247–249	249–250
3	4-OCH_3_	C_6_H_5_	**5c**	3 h	90	180–182	181–183
4	4-CH_3_	C_6_H_5_	**5d**	2.5 h	90	188–190	189–190
5	4-Cl	-CH_2_C_6_H_5_	**5e**	3 h	89	166–168	163–165
6	4-OH	-CH_2_C_6_H_5_	**5f**	3 h	80	132–134	131–132
7	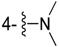	-CH_2_C_6_H_5_	**5g**	3.5 h	73	154–155	155–157
8	4-CH_3_	-CH_2_C_6_H_5_	**5h**	3.5 h	75	160–162	163–165
9	3-OCH_3_	-CH_2_C_6_H_5_	**5i**	4 h	70	115–117	128–129
10	3,4-OCH_3_	-CH_2_C_6_H_5_	**5j**	4 h	68	189–191	188–190

^a^ without use of solvent, neat. ^b^ Isolated yield.

### 2.2. Reusability of the Catalyst

Based on the above results and following the reaction optimization, we decided to explore the recyclability of β-CD-PSA taking the synthesis of **5a** as a model reaction (Table 4). The catalyst was easily recovered upon filtration of the reaction mixture after addition of water. The filtrates were dried under vacuum and the resulting catalyst was reused directly for the next run. It has been found that the catalyst can be efficiently recycled for four consecutive runs with slight loss of activity. It was predicted that the catalyst recovery was efficient with a gradual decline in yield of product.

**Table 4:** Reusability of β-CD-PSA as catalyst for **5a**.

No. of Runs	Percentage Yield
1	96
2	94
3	90
4	90

### 2.3. Pharmacology

#### Local Anesthetic Activity

The target compounds were subjected to evaluation of local anesthetic activity on rabbit corneal and mouse tail anesthetic models which mimic surface and infiltration anesthesia. As shown in Table 5, except for compounds **5g** and **5i**, none of the derivatives showed any significant activity in both the models. Compound **5g** found to be more active than lidocaine used as a standard. In surface anesthesia, more than half the maximal response was observed in the case of compound **5h**. We can summarized that the presence of a benzylamine group has significant impact on the anesthetic potential of the compounds, while its replacement by aniline makes the compounds inactive.

**Table 5:** Local anesthetic evaluation of the target compounds in the rabbit corneal and mouse tail anesthetic models.

Compound	Corneal Anesthesia ^a^	Mouse Tail Anesthesia ^b^
**5a**	20.3 ± 8.6	11.3 (±0.11) × 10^−2^
**5b**	12.4 ± 9.3	5.7 (±0.42) × 10^−2^
**5c**	15.5 ± 12.3	6.5 (±0.23) × 10^−2^
**5d**	10.32 ± 0.12	4.3 (±0.54) × 10^−2^
**5e**	21.65 ± 4.7	3.4 (±0.32) × 10^−2^
**5f**	5.5 ± 3.5	5.4 (±0.14) × 10^−2^
**5g**	121.23 ± 12.3	1.6 (±0.26) × 10^−2^
**5h**	65.65 ± 5.8	9.2 (±0.16) × 10^−2^
**5i**	95.4 ± 13.2	7.4 (±0.18) × 10^−2^
**5j**	20.3 ± 10.3	4.1 (±0.45) × 10^−2^
Lidocaine HCl ^c^	100	2.1 (±0.25) × 10^−2^

^a^ All compounds were in aqueous solution at 2% concentration. The values, expressed as percentage of the anesthetic activity of lidocaine (=100), are means ± SE of three determinations. ^b^ IC_50_ values expressed as mol/L. ^c^ Lidocaine hydrochloride was used as standard agent for comparison.

**Figure 1:**
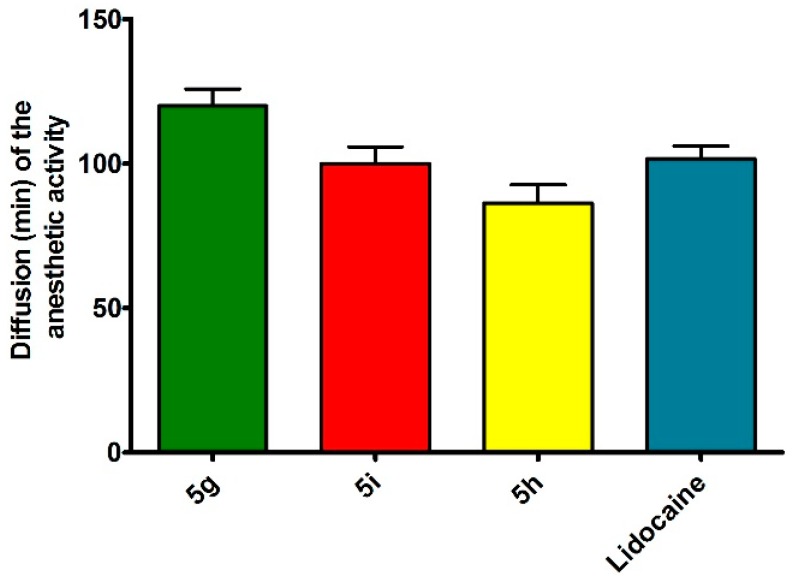
*In vivo* duration of local anesthetic activity (rat sciatic nerve block) of lidocaine and compounds **5g**, **5i** and **5h**. Each rat received 0.2 mL of the 2% anesthetic solution. The values are means ± standard deviation of three independent experiments.

Owing to the excellent anesthetic activity, these compounds were further subjected to a rat sciatic nerve block assay for the determination of the duration of anesthetic activity. The result is shown Figure 1. It has been found that compound **5g** exhibits a similar anesthetic profile to that of the reference drug lidocaine.

**Figure 2:**
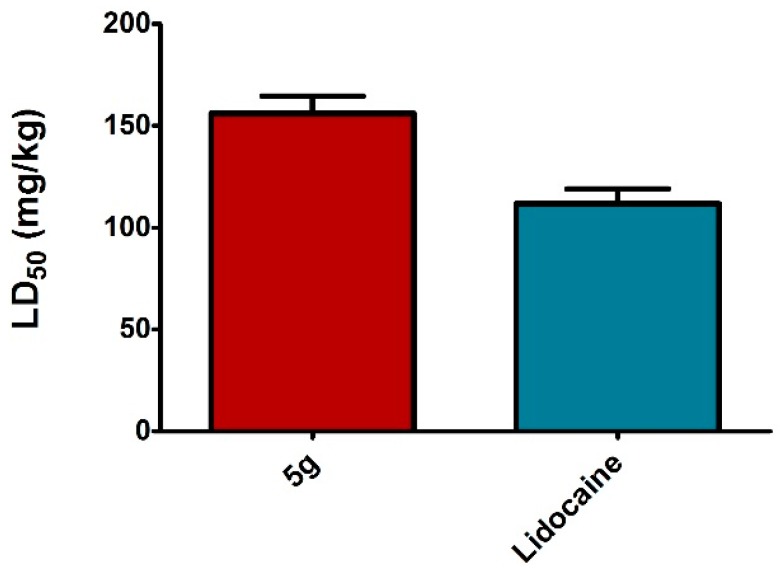
Acute toxicity of (ip) of lidocaine and compound **5g** in male mice.

**Figure 3:**
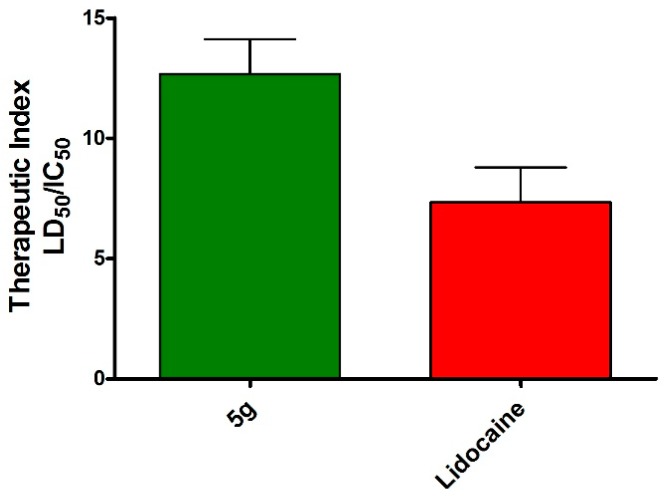
Therapeutic index of lidocaine and compound **5g** evaluated as ratio between LD_50_ and IC_50_ (mouse tail test expressed in mg/kg).

The compound **5g** was further subjected for the determination of the acute toxicity and therapeutic index in mice. As shown in Figure 2 (LD_50_) and Figure 3 (LD_50_/IC_50_), it is characterised by the most favourable ratio between toxicity and anesthetic activity. Moreover, the therapeutic index of the above compound was indicated as significantly higher than that of the lidocaine standard.

## 3. Experimental Section

### 3.1. General Information

All chemicals were of analytical grade and used directly. Melting points were determined in open capillary tubes with an Electrothermal melting point apparatus and are uncorrected. The completion of reaction was checked by thin layer chromatography (TLC) using silica gel-G coated Al-plates (0.5 mm thickness; Merck, Darmstadt, Germany) and the plates were illuminated under UV (254 nm) and evaluated in iodine vapour. FT-IR (in 2.0 cm^−1^, flat, smooth, abex, KBr) spectra were recorded on a Spectrum RX-I spectrometer (Perkin Elmer Instruments, Buckinghamshire, UK). Elemental analysis were determined on a Vario EL III CHNOS elemental analyzer (Elementar, Hanau, Germany). ^1^H-NMR spectra were recorded on a Bruker Avance II 400 NMR Spectrometer and the ^13^C-NMR spectra on a Bruker Avance II 100 NMR spectrometer (Bruker Corporation, Billerica, MA, USA) in D_2_O or DMSO-*d*_6_ using TMS as the internal standard. The chemical shifts are reported in parts per million (ppm, d), and signals are described as s (singlet), d (doublet), t (triplet), q (quartet), and m (multiplet). Mass spectra were obtained on a VG-AUTOSPEC spectrometer (VG Analytical, Manchester, UK).

#### 3.1.1. Synthesis of β-CD-PSA

Initially, the β-CD (1 g) was dissolved in NaOH solution (5 M, 10 mL) at 75 °C. To the above solution, 1,3-propane sultone (1.1 g) was added in small amounts and the mixture was stirred for 4 h. The reaction solution was then cooled to room temperature and adjusted to neutral using HCl solution (3 M). The mixture was poured in ethanol to afford sulfopropyl ether β-cyclodextrin (SPE-β-CD). In the next step, the acidic resin was activated in a saturated aqueous solution of NaCl for 1 day. This was followed by treatment with 2.5 wt % NaOH aqueous solution for 80 min, and then washing with distilled water until the pH reaches neutral. It was treated further with 5.0 wt % HCl aqueous solution for 12 h and afterwards, the resin was transferred to a column and washed with deionized water until the eluent become neutral.

Finally, the sodium salt of SPE-β-CD (0.5 g) was dissolved in water (100 mL), and the solution was allowed to flow through the acidic resin column at a speed of 20 drops per minute. The acidic eluent was collected and then freeze-dried for 12 h to obtain the β-CD-PSA product. FT-IR (ν_max_; cm^−1^ KBr): 3354, 2930, 2365, 1653, 1361, 1153, 1030, 700. ^1^H-NMR (400 MHz, CDCl_3_) δ, ppm: 5.01–5.11 (m, C1–H), 3.49–3.81 (m, –OCH_2_CH_2_CH_2_SO_3_H, CH), 2.85 (s, –OCH_2_ CH_2_CH_2_SO_3_H), 2.28 (s, –OCH_2_CH_2_CH_2_SO_3_H); ^13^C-NMR (100 MHz, D_2_O): δ, ppm: 80.9, 72.7, 71.6, 69.4, 67.8, 53.2, 28.2, 27.8, 21.1, 19.9; Anal. Found: C 33.52, S 7.834, H 6.67.

#### 3.1.2. General Procedure for the Synthesis of 1,2,4,5-Tetrasubstituted Imidazoles **5**(**a**–**j**)

The catalyst β-CD-PSA (0.02 mol), an aromatic aldehyde (1 mmol), benzil (1 mmol), aniline or benzylamine (1 mmol) and ammonium acetate (3 mmol) were placed in a flask and stirred at 100 °C under solvent free conditions for a suitable time (Table 3). The progress of the reaction was monitored by TLC. After completion of the reaction, ethyl acetate (5 mL) was added to the reaction mixture, which was filtered to remove the insoluble catalyst. The crystals of 1,2,4,5-tetrasubstituted imidazoles appeared after gradual evaporation of solvent at room temperature. The solution of β-CD-PSA was dried under vacuum for recycling the catalyst in the next run, and can be used several times without much loss of catalytic activity.

*2-(4-Chlorophenyl)-1,4,5-triphenyl-1H-imidazole* (**5a**). MW: 406.91; R*_f_*: 0.75; FT-IR (ν_max_; cm^−1^ KBr): 3038, 2979 (C-H_stretch_), 1648 (C=N_stretch_), 1215 (C-N_stretch_), 891 (aromatic-Cl) cm^−1^; ^1^H-NMR (400 MHz, CDCl_3_) δ, ppm: 8.09–8.04 (d, 2H *J* = 8.4 Hz, Ar-H), 7.79–7.76 (d, 2H *J* = 8.1 Hz, Ar-H), 7.58–7.41 (m, 15H, Ar-H); ^13^C-NMR (100 MHz, CDCl_3_) δ, ppm: 144.2, 141.3, 138.8, 138.2, 134.3, 133.1, 131.8, 129.7, 129.3, 129.2, 128.9, 128.7, 128.2, 127.5, 122.1; Mass spectrum: 407.89 (M + 1); Elemental analysis for C_27_H_19_ClN_2_: Calculated: C, 79.70; H, 4.71; N, 6.88; Found: C, 79.68; H, 4.71; N, 6.89.

*2-(3-Nitrophenyl)-1,4,5-triphenyl-1H-imidazole* (**5b**). MW: 417.46; R*_f_*: 0.63; FT-IR (ν_max_; cm^−1^ KBr): 3045, 2972 (C-H_stretch_), 1651 (C=N_stretch_), 1213 (C-N_stretch_), 878 (aromatic-NO_2_) cm^−1^; ^1^H-NMR (400 MHz, CDCl_3_) δ, ppm: 8.58–8.53 (d, 2H *J* = 8.2 Hz, Ar-H), 7.94–7.91 (d, 2H *J* = 7.6 Hz, Ar-H), 7.73–7.42 (m, 15H, Ar-H); ^13^C-NMR (100 MHz, CDCl_3_) δ, ppm: 148.5, 144.4, 138.7, 138.2, 133.7, 133.1, 131.7, 131.4, 130.2, 129.6, 129.2, 128.7, 128.2, 127.5, 123.9, 122.8, 122.1; Mass spectrum: 418.48 (M + 1); Elemental analysis for C_27_H_19_N_3_O_2_: Calculated: C, 77.68; H, 4.59; N, 10.07; Found: C, 77.69; H, 4.61; N, 10.08.

*2-(4-Methoxyphenyl)-1,4,5-triphenyl-1H-imidazole* (**5c**). MW: 402.49; R*_f_*: 0.78; FT-IR (ν_max_; cm^−1^ KBr): 3052, 2971 (C-H_stretch_), 1653 (C=N_stretch_), 1218 (C-N_stretch_), 892 cm^−1^; ^1^H-NMR (400 MHz, CDCl_3_) δ, ppm: 7.98–7.94 (d, 2H *J* = 8.3 Hz, Ar-H), 7.79–7.41 (m, 15H, Ar-H), 7.04–7.01 (d, 2H *J* = 6.3 Hz, Ar-H), 3.87 (s, 3H, OCH_3_); ^13^C-NMR (100 MHz, CDCl_3_) δ, ppm: 160.6, 144.4, 138.7, 138.2, 135.5, 133.1, 131.7, 130.4, 129.7, 129.2, 128.7, 128.1, 127.5, 122.2, 114.8, 55.9; Mass spectrum: 403.49 (M + 1); Elemental analysis for C_28_H_22_N_2_O: Calculated: C, 83.56; H, 5.51; N, 6.96; Found: C, 83.58; H, 5.52; N, 6.97.

*1,4,5-Triphenyl-2-(p-tolyl)-1H-imidazole* (**5d**). MW: 386.49; R*_f_*: 0.65; FT-IR (ν_max_; cm^−1^ KBr): 3059, 2968 (C-H_stretch_), 1657 (C=N_stretch_), 1214 (C-N_stretch_), 899 cm^−1^; ^1^H-NMR (400 MHz, CDCl_3_) δ, ppm: 8.58–8.56 (d, 2H *J* = 8.4 Hz, Ar-H), 7.78–7.43 (m, 15H, Ar-H), 7.28–7.24 (d, 2H *J* = 6.7 Hz, Ar-H), 2.35 (s, 3H, CH_3_); ^13^C-NMR (100 MHz, CDCl_3_) δ, ppm: 144.5, 140.2, 138.7, 138.2, 133.1, 131.7, 129.7, 129.4, 129.1, 128.7, 128.1, 127.5, 125.7, 122.7, 21.3; Mass spectrum: 387.51 (M + 1); Elemental analysis for C_28_H_22_N_2_: Calculated: C, 87.01; H, 5.74; N, 7.25; Found: C, 87.03; H, 5.74; N, 7.26.

*1-Benzyl-2-(4-chlorophenyl)-4,5-diphenyl-1H-imidazole* (**5e**). MW: 420.93; R*_f_*: 0.64; FT-IR (ν_max_; cm^−1^ KBr): 3063, 2969 (C-H_stretch_), 1654 (C=N_stretch_), 1211 (C-N_stretch_), 893 (Cl) cm^−1^; ^1^H-NMR (400 MHz, CDCl_3_) δ, ppm: 8.12–8.09 (d, 2H *J* = 8.2 Hz, Ar-H), 7.78–7.23 (m, 15H, Ar-H), 7.48–7.44 (d, 2H *J* = 6.2 Hz, Ar-H), 5.59 (s, 2H, CH_2_); ^13^C-NMR (100 MHz, CDCl_3_) δ, ppm: 153.7, 141.4, 138.2, 137.3, 134.3, 133.1, 129.6, 129.4, 129.1, 128.9, 128.4, 128.2, 127.6, 127.5, 125.7, 47.5; Mass: 421.94 (M + 1); Elemental analysis for C_28_H_21_ClN_2_: Calculated: C, 79.89; H, 5.03; N, 6.66; Found: C, 79.91; H, 5.03; N, 6.66.

*4-(1-Benzyl-4,5-diphenyl-1H-imidazol-2-yl)phenol* (**5f**). MW: 402.49; R*_f_*: 0.51; FT-IR (ν_max_; cm^−1^ KBr): 3068, 2962 (C-H_stretch_), 1657 (C=N_stretch_), 1218 (C-N_stretch_), 897 cm^−1^; ^1^H-NMR (400 MHz, CDCl_3_) δ, ppm: 7.91–7.89 (d, 2H *J* = 8.4 Hz, Ar-H), 7.79–7.23 (m, 15H, Ar-H), 6.89–6.84 (d, 2H *J* = 6.7 Hz, Ar-H), 5.58 (s, 2H, CH_2_), 5.28 (s, 1H, OH); ^13^C-NMR (100 MHz, CDCl_3_) δ, ppm: 158.5, 153.7, 141.3, 138.2, 137.3, 133.1, 130.7, 129.6, 129.1, 128.8, 128.4, 127.6, 127.3, 125.7, 123.2, 116.4, 47.5; Mass spectrum: 403.51 (M + 1); Elemental analysis for C_28_H_22_N_2_O: Calculated: C, 83.56; H, 5.51; N, 6.96; Found: C, 83.58; H, 5.52; N, 6.96.

*4-(1-Benzyl-4,5-diphenyl-1H-imidazol-2-yl)-N,N-dimethylaniline* (**5g**). MW: 429.56; R*_f_*: 0.61; FT-IR (ν_max_; cm^−1^ KBr): 3069, 2957 (C-H_stretch_), 1652 (C=N_stretch_), 1215 (C-N_stretch_), 891 cm^−1^; ^1^H-NMR (400 MHz, CDCl_3_) δ, ppm: 7.97–7.95 (d, 2H *J* = 8.2 Hz, Ar-H), 7.79–7.24 (m, 15H, Ar-H), 6.82–6.79 (d, 2H *J* = 6.4 Hz, Ar-H), 5.58 (s, 2H, CH_2_), 3.06 (s, 6H, CH_3_ × 2); ^13^C-NMR (100 MHz, CDCl_3_) δ, ppm: 155.3, 153.7, 141.3, 138.2, 137.3, 133.1, 129.6, 129.2, 128.7, 128.4, 128.1, 127.6, 127.2, 125.7, 120.2, 112.8, 47.5, 41.3; Mass spectrum: 430.58 (M + 1); Elemental analysis for C_30_H_27_N_3_: Calculated: C, 83.88; H, 6.34; N, 9.78; Found: C, 83.91; H, 6.33; N, 9.78.

*1-Benzyl-4,5-diphenyl-2-(p-tolyl)-1H-imidazole* (**5h**). MW: 400.51; R*_f_*: 0.53; FT-IR (ν_max_; cm^−1^ KBr): 3069, 2956 (C-H_stretch_), 1659 (C=N_stretch_), 1218 (C-N_stretch_), 887 cm^−1^; ^1^H-NMR (400 MHz, CDCl_3_) δ, ppm: 8.48–8.46 (d, 2H *J* = 8.4 Hz, Ar-H), 7.79–7.22 (m, 15H, Ar-H), 7.28–7.24 (d, 2H *J* = 6.1 Hz, Ar-H), 5.58 (s, 2H, CH_2_), 2.28 (s, 3H, CH_3_); ^13^C-NMR (100 MHz, CDCl_3_) δ, ppm: 153.7, 141.3, 138.2, 137.3, 133.1, 131.7, 129.8, 129.5, 129.1, 128.7, 128.3, 127.6, 127.2, 125.7, 47.5, 21.3; Mass spectrum: 401.48 (M + 1); Elemental analysis for C_29_H_24_N_2_: Calculated: C, 86.97; H, 6.04; N, 6.99; Found: C, 86.98; H, 6.04; N, 6.98.

*1-Benzyl-2-(4-methoxyphenyl)-4,5-diphenyl-1H-imidazole* (**5i**). MW: 416.51; R*_f_*: 0.65; FT-IR (ν_max_; cm^−1^ KBr): 3069, 2959 (C-H_stretch_), 1655 (C=N_stretch_), 1216 (C-N_stretch_), 884 cm^−1^; ^1^H-NMR (400 MHz, CDCl_3_, TMS) δ, ppm: 7.97–7.96 (d, 2H *J* = 8.3 Hz, Ar-H), 7.79–7.23 (m, 15H, Ar-H), 7.05–7.02 (d, 2H *J* = 6.4 Hz, Ar-H), 5.58 (s, 2H, CH_2_), 3.82 (s, 3H, OCH_3_); ^13^C-NMR (100 MHz, CDCl_3_) δ, ppm: 160.6, 153.7, 141.2, 138.3, 137.3, 133.1, 130.2, 129.7, 129.2, 128.8, 128.3, 127.8, 127.4, 125.6, 122.8, 114.8, 55.7, 47.5; Mass spectrum: 417.51 (M + 1); Elemental analysis for C_29_H_24_N_2_O: Calculated: C, 83.63; H, 5.81; N, 6.73; Found: C, 83.64; H, 5.80; N, 6.73.

*1-Benzyl-2-(3,4-dimethoxyphenyl)-4,5-diphenyl-1H-imidazole* (**5j**). MW: 446.54; R*_f_*: 0.69; FT-IR (ν_max_; cm^−1^ KBr): 3073, 2955 (C-H_stretch_), 1659 (C=N_stretch_), 1218 (C-N_stretch_), 881 cm^−1^; ^1^H-NMR (400 MHz, CDCl_3_) δ, ppm: 7.53–7.39 (d, 2H *J* = 8.7 Hz, Ar-H), 7.79–7.24 (m, 15H, Ar-H), 6.94 (s, 1H, Ar-H), 5.57 (s, 2H, CH_2_), 3.83 (s, 6H, OCH_3_ × 2); ^13^C-NMR (100 MHz, CDCl_3_) δ, ppm: 153.7, 150.3, 149.8, 141.3, 138.2, 137.3, 133.1, 129.6, 129.1, 128.8, 128.4, 127.6, 127.3, 125.7, 123.9, 122.3, 112.3, 111.4, 56.2, 47.6; Mass spectrum: 447.56 (M + 1); Elemental analysis for C_30_H_26_N_2_O_2_: Calculated: C, 80.69; H, 5.87; N, 6.27; Found: C, 80.71; H, 5.88; N, 6.27.

### 3.2. Pharmacology

#### 3.2.1. Corneal Anesthesia

Male New Zealand rabbits were used for the determination of local anesthetic activity of the synthesized compounds. Local surface anesthesia was evaluated by determining the number of stimuli to the cornea every 3 min. [25] This was affected rhythmically with a Frey’s horse-hair, in order to produce the blink reflex. If the reflex did not occur after 100 stimulations, anesthesia was considered total. At the beginning of the experiment, special care was taken to ascertain that this reflex was normal in both eyes of the rabbits used. The aqueous solutions (2%) of the compounds studied were dropped onto the conjunctival sac so that the space between the eyelids contained a clearly visible film of solution for the set time of 3 min. Lidocaine solution (2%) was used as standard for comparison.

#### 3.2.2. Mouse Tail Anesthesia

Male Swiss mice (weighing 18–20 g) were used. The test was performed according to the method of Bianchi in which the aqueous anesthetic solution (0.1 mL) is injected SC about 1 cm from the root of the tail [26]. Fifteen minutes after injection, the pain reflex of all the injected animals was tested applying a small artery clip to the zone where the compound was injected. The proportion of animals which do not show the usual pain reflex within 30 s was noted for each dose. Lidocaine solutions were used for comparison. IC_50_ values were calculated for each compound by probit analysis using a computer program [27].

#### 3.2.3. Rat Sciatic Nerve Block

Triplicate sets of three groups of three male Wistar rats (weighing 180–200 g) were used, according to the method as outlined by Al-Saadi and Sneader [28]. Each rat received an injection (0.2 mL) of the aqueous anesthetic solution (2%) into the posterior aspect of the femur head. A positive effect of the drug resulted in a complete loss of motor control of the injected limb. In order to assess the duration of the effect, the animals were observed from the time of onset of the motor paralysis at 5 min intervals for the first 30 min, and at 15 min intervals after that up to the first sign of motor activity.

#### 3.2.4. Acute Toxicity

The intra paritoneal acute toxicity of the most active compound **5g** was determined in male Swiss mice weighing 18–20 g 7 days after treatment. LD_50_ values were calculated for each compound by probit analysis using a computer program [27].

## 4. Conclusions

In summary, we have presented a novel, highly rapid and efficient protocol for the synthesis of 1,2,4,5-tetrasubstituted imidazoles as local anesthetic agents via a one-pot condensation reaction. The developed methodology offers an environmentally benign and safe protocol which includes a simple reaction setup not requiring specialized equipment, and all the reactions peform well at 100 °C to afford the desired compounds in excellent yield without use of the solvent. Moreover, the catalyst used in present method can be recovered and recycled making the procedure potentially useful for commercial applications. In bioactivity screening using various methods, some of these compounds were identified as excellent potential local anesthetic agents. Thus, together with efficient methodology of synthesis and pronounced local anesthetic activity, these compounds may serve as a prospective leads for further drug development.
